# Genome Sequence of Hymenobacter polaris RP-2-7^T^, Isolated from Arctic Soil

**DOI:** 10.1128/MRA.01216-20

**Published:** 2021-01-07

**Authors:** Aravind Sundararaman, Ram Hari Dahal, Dong-Uk Kim, Jaisoo Kim, Jitendra Upadhyaya, Yongseok Hong, Dhiraj Kumar Chaudhary

**Affiliations:** a Department of Microbiology and Fermentation Technology, Central Food Technological Research Institute (CSIR), Mysuru, India; b Department of Microbiology, School of Medicine, Kyungpook National University, Daegu, Republic of Korea; c Department of Biological Science, College of Science and Engineering, Sangji University, Wonju, Republic of Korea; d Department of Life Science, College of Natural Sciences, Kyonggi University, Suwon, Kyonggi-Do, Republic of Korea; e Department of Bioresource Engineering, McGill University, Ste-Anne-de-Bellevue, Montreal, Quebec, Canada; f Department of Environmental Engineering, Korea University Sejong Campus, Sejong City, South Korea; Broad Institute

## Abstract

Hymenobacter polaris RP-2-7^T^ was isolated from soil from the Arctic region. This study presents the genome sequence of *Hymenobacter polaris* RP-2-7^T^, generated using the Illumina HiSeq platform. The genome size is 5,587,174 bp; it contains 4,721 genes and has 62.8 mol% DNA G+C content.

## ANNOUNCEMENT

Hymenobacter polaris is a Gram-negative, non-spore-forming, strictly aerobic, rod-shaped, oxidase- and catalase-positive bacterium isolated from soil at an Arctic station located at Spitsbergen, Svalbard, Norway (8°053′22.8″N, 12°09′12.9″E). Colonies grown on Reasoner’s 2A (R2A) agar are brilliant pink, entire, convex, and circular (1). *H. polaris* RP-2-7^T^ is a psychrotolerant bacterium that optimally grows at 15 to 20°C (1). For whole-genome sequencing (WGS), strain RP-2-7^T^ colonies were grown on R2A agar plates at 20°C for 3 days, and genomic DNA was extracted using DNeasy blood and tissue kits (Qiagen). Whole-genome shotgun sequencing was accomplished at Macrogen Laboratory. The sequencing libraries were generated with the KAPA HyperPrep kit (catalog number KK8504) and sequenced using the Illumina HiSeq 2500 platform using a 2 × 150-bp paired-end kit, which produced a total of 3,780,270 paired-end reads. Trimmomatic v0.36 was used to remove adapter sequences (2), and the obtained sequence was assembled by A5-miseq (3). Default parameters were used for all software. The genome sequence was annotated using the NCBI Prokaryotic Genome Annotation Pipeline (PGAP) v8.0 (4) and the Rapid Annotations using Subsystems Technology (RAST) server v2.0 (5). A circular genomic feature map was constructed using CGView Server^BETA^ (6). The WGS of strain RP-2-7^T^ was 5,587,174 bp long with 8 contigs, an *N*_50_ value of 1,470,772 bp, and 233.0× genome coverage. The G+C content of the genome sequence was 62.8 mol%. A total of 4,721 genes, 4,614 protein-coding genes, 7 rRNAs, 42 tRNAs, and 55 pseudogenes were predicted. The *H. polaris* genome contained coding sequences (CDSs) for cold shock proteins that are known to be associated with adaptation to cold temperatures (GenBank accession numbers NML64068, NML64069, NML66199, NML67535, and NML67536). Carbon storage-related genes and the glycogen-debranching gene (*glgX*) (NML64394 and NML65075) were found in the genome sequence. In addition, a glycogen/starch synthase enzyme (NML66870) was also present. These genes help the microorganism to adapt during growth in cold environments (7).

The genome sequence also contained several cAMP metabolism-related genes, such as the cyclic AMP receptor protein (FNR; GenBank accession numbers NML63690, NML65182, NML65548, NML65757, NML65999, NML66099, NML66297, NML67898, and NML67987), cAMP-dependent protein kinases (CGA), and the cAMP-dependent Kef type K^+^ transport system (AKT). The genome contained multidrug efflux pumps of the RND (resistance, nodulation, division) family, including the genes *cmeA*, *cmeB*, and *cmeC*, transcription regulators of the *TetR* family (NML65257, NML65313, NML65638, NML65729, and NML66061), and the macrolide-specific efflux gene *macA*. Gene annotation using the RAST server identified the presence of 265 subsystems for the *H. polaris* genome sequence. Annotation and analysis of the secondary metabolite biosynthesis genes using anti-SMASH v5.0 (8) revealed that *H. polaris* contains a nonribosomal peptide synthetase (NRPS)-like enzyme, NRPS-type I polyketide synthase (T1PKS), terpene, and type III polyketide synthase (T3PKS), as well as the most similar known gene cluster of carotenoids. This *H. polaris* genome sequence will provide an understanding of the genomic diversity of *H. polaris* strains and also help us to understand cold adaption by psychrotolerant species.

### Data availability.

This whole-genome shotgun project has been deposited in DDBJ/ENA/GenBank under the accession number JABBGH00000000. The raw sequencing reads are available at the Sequence Read Archive (SRA) under accession number SRR13039948 and are associated with BioProject accession number PRJNA626506.
